# Age Friendly, but Not for Me? Resolving Cognitive Dissonance to Improve Continuity of Care

**DOI:** 10.1093/geroni/igaf122.3152

**Published:** 2025-12-31

**Authors:** Joshua Gerlick, Betty Napoleon, Grace Armstrong, Nicholas Schiltz, Anne Pohnert, Mary Dolansky

**Affiliations:** Case Western Reserve University, Cleveland, Ohio, United States; Case Western Reserve University, Cleveland, Ohio, United States; Case Western Reserve University, Cleveland, Ohio, United States; Case Western Reserve University, Cleveland, Ohio, United States; CVS Health, Woonsocket, Rhode Island, United States; Case Western Reserve University, Cleveland, Ohio, United States

## Abstract

A persistent challenge in geriatric healthcare is ensuring that older adults effectively transition from Age-Friendly Health Systems (AFHS) care provided at retail clinics to ongoing follow-up with their primary care providers (PCPs). We conducted three focus groups (N = 20, ages 64–81) to understand older adults’ experiences receiving care through the AFHS 4Ms framework at MinuteClinics in select CVSHealth locations, as well as the barriers and facilitators influencing their decisions to follow up with a PCP. Using a constructivist grounded theory approach, we applied team-based initial (open) coding techniques to capture participants’ lived experiences during visits to a MinuteClinic. Axial coding then identified deeper relational themes, most notably cognitive dissonance and information uncertainty. Participants frequently expressed contradictory views on the relevance of AFHS, describing it as beneficial in general yet personally irrelevant. One participant captured this tension by stating, “This doesn’t apply to me, but it’s valuable for others with more serious conditions.” We supported these findings with members of the CVS MinuteClinic AFHS Patient Advisory Council (PAC), which highlighted identity-related discomfort with aging. One PAC member remarked, “We don’t think of ourselves as old,” and this creates a barrier to embracing AFHS recommendations. Focus group and PAC participants reported uncertainty about how to best follow-up and integrate AFHS recommendations with their PCP, suggesting a need for clearer and more personalized messaging. Our findings suggest that providers must explicitly link AFHS 4Ms recommendations with patients’ individual health contexts to reduce cognitive dissonance, enhance clarity, and improve the likelihood of follow-up.

